# Transcriptomic landscape of *Pueraria lobata* demonstrates potential for phytochemical study

**DOI:** 10.3389/fpls.2015.00426

**Published:** 2015-06-22

**Authors:** Rongchun Han, Hiroki Takahashi, Michimi Nakamura, Naoko Yoshimoto, Hideyuki Suzuki, Daisuke Shibata, Mami Yamazaki, Kazuki Saito

**Affiliations:** ^1^Department of Molecular Biology and Biotechnology, Graduate School of Pharmaceutical Sciences, Chiba UniversityChiba, Japan; ^2^Pharmacy College, Liaoning University of Traditional Chinese MedicineDalian, China; ^3^Medical Mycology Research Center, Chiba UniversityChiba, Japan; ^4^Kazusa DNA Research InstituteChiba, Japan

**Keywords:** *Pueraria lobata*, Leguminosae, Kudzu, deep-transcriptome analysis

## Abstract

*Pueraria lobata* (Willd.) Ohwi has a long and broad application in the treatment of disease. However, in the US and EU, it is treated as a notorious weed. The information to be gained from decoding the deep transcriptome profile would facilitate further research on *P. lobata*. In this study, more than 93 million fastq format reads were generated by Illumina’s next-generation sequencing approach using five types of *P. lobata* tissue, followed by CLC *de novo* assembly methods, ultimately yielding about 83,041 contigs in total. Then BLASTx similarity searches against the NCBI NR database and UniProtKB database were conducted. Once the duplicates among BLASTx hits were eliminated, ID mapping against the UniProt database was conducted online to retrieve Gene Ontology information. In search of the putative genes relevant to essential biosynthesis pathways, all 1,348 unique enzyme commission numbers were used to map pathways against the Kyoto Encyclopedia of Genes and Genomes. Enzymes related to the isoflavonoid and flavonoid biosynthesis pathways were focused for detailed investigation and subsequently, quantitative real-time reverse transcription polymerase chain reaction was conducted for biological validation. Metabolites of interest, puerarin and daidzin were studied by HPLC. The findings in this report may serve as a footstone for further research into this promising medicinal plant.

## Introduction

*Pueraria lobata* (Willd.) Ohwi (Kudzu) has been described and used as a traditional medicinal plant for more than 20 centuries in oriental medicine (Keung and Vallee, 1998). The *P. lobata* root, a part of the plant that is prescribed most frequently, accumulates abundant polyphenolic compounds, including isoflavones, isoflavonoid glycosides, coumarins, puerarols and the associated derivatives (Wong et al., 2011). Intensive investigation has revealed a chemical profile with antioxidant and antimutagenic activity (Miyazawa et al., 2001; Cherdshewasart and Sutjit, 2008) and efficacy in the treatment of alcoholism (Carai et al., 2000) and diabetic retinopathy (Cherdshewasart et al., 2007; Teng et al., 2009). The plant was introduced to the United States in 1876 as an ornamental plant and then to Europe. Due to its rapid growth and vigorous adaptation to the surroundings, *P. lobata* is now regarded as a major ecosystem threat (Follak, 2011) and a noxious weed, according to the USDA plant database. In order to evaluate the plant’s potential as a cure for disease or regulate its invasive influence on other native plants, additional research using next-generation sequencing technologies into this leguminous plant is necessary.

The genomes for many model organisms have been sequenced but for these non-model plants, the lack of reference genome information hinders studies on the underlying genes involved in essential biological processes related to drug development. In this regard, transcriptomic sequencing plays an essential role in understanding the genetic diversity across organisms. Such approaches elucidate the genetic code that underlies protein diversity (Muranaka and Saito, 2013; Saito, 2013). The use of new technologies such as the Short Oligonucleotide Analysis Package (SOAPdenovo; Li et al., 2009), Assembly by Short Sequences (AbySS; Simpson et al., 2009), and Trinity (Grabherr et al., 2011) accelerates the pace of transcriptomic profiling when processing tremendous amounts of data generated from large-scale sequencing projects. Massively parallel cDNA sequencing (RNA-Seq) measures the levels of transcripts and their isoforms far more precisely than other methods (Fullwood et al., 2009; Wang et al., 2009).

*C*-glycosides are widespread in plants, insects and microbes, where they serve a diverse range of functions including acting as antibiotics, antioxidants, attractants, and feeding deterrents (Brazier-Hicks et al., 2009). Early study on *Fagopyrum esculentum* seedlings showed 2-hydroxylation of flavanones was a critical prerequisite for the corresponding *C*-glucosyltransferase to catalyze (Kerscher and Franz, 1987). Recently, reports regarding *C*-glycosylation of flavonoids in crops such as wheat and corn suggested considerable similarity of the proteins some of which exhibited bifunctional *C*- and *O*-glucosyltransferase activity (Ferreyra et al., 2013). For the characteristic compound daidzein-8-*C*-glycoside (puerarin) found in *P. lobata*, although the biosynthetic pathway for daidzein in legumes is well established (Steele et al., 1999; Jung et al., 2000), the key enzyme responsible for catalyzing this isoflavone aglycon remains to be identified.

In this study, 93,248,914 paired-end reads of *P. lobata* were generated from five different tissues by Illumina’s sequencing platform. Illumina reads were deposited at the DDBJ Sequence Read Archive (DRA) with accession number (AC) DRA001736 and the resultant contigs along with the top hits of BLASTx at GitHub^1^. CLC Genomics Workbench (CLC bio, Aarhus, Denmark) was subsequently applied to conduct *de novo* assembly. Based on the findings provided by Gene Ontology (GO) and KEGG pathway mapping, the candidate genes that may be involved in the biosynthesis of key chemical components were identified (Han et al., 2015). For biological validation, quantitative real-time reverse transcription polymerase chain reaction (qRT-PCR) was applied to check the genuine expression profile for the genes involved in the biosynthetic pathway leading to isoflavonoids. Meanwhile, the concentrations of puerarin and daidzin, the characteristic compounds in Kudzu, were measured by High Performance Liquid Chromatography (HPLC) within the five tissues from which we obtained the deep transcriptomic data.

## Materials and Methods

### Plant Materials, Chemicals and Total RNA Extraction

The basic technology of RNA extraction, RNA-seq analysis and informatics is similar as described elsewhere (Han et al., 2015). Fresh tissues and organs were collected from healthy *P. lobata* plants growing in Chiba, Japan in May 2012. Puerarin and daidzin standard substances were purchased from LC laboratories (USA). The materials were kept in RNA stabilization solution (RNA*later*, Life technologies, USA) immediately after sampling. The RNA*later* solution was gently removed with a Kimwipe, and the remaining sample was frozen by liquid nitrogen and powdered using Multi Beads Shocker (Yasui Kikai, Japan). TRIzol Reagent (Invitrogen, USA) was used to extract total RNA from powdered *P. lobata*. The RNA obtained was then treated using the RNeasy Mini Kit (Qiagen, USA).

### cDNA Library Preparation and Sequencing

The TruSeq RNA Sample Prep Kit v2 (Illumina, CA, USA) was used for cDNA library preparation and sequencing. Once the mRNA in total RNA had been polyA-selected and fragmented, double-stranded cDNA was prepared for cDNA library construction. After the creation of blunt-end fragments and indexed adaptor ligation, the samples were hybridized to flow cells. Cluster amplification was completed using the cBot Cluster Generation System (Illumina, CA, USA) and then sequenced by Illumina’s next-generation sequencing instrument, the HiSeq 1000 as described (Han et al., 2015).

### CLC Approach to *De Novo* Assembly and Transcript Abundance Analysis

Prior to assembly, the original fastq *P. lobata* format data were subjected to CLC trimming to eliminate reads of poor quality. The CLC method (version 4.9) was used to process clean reads. The publically available *P. lobata* expressed sequence tags (ESTs) data (6,365) were downloaded from the National Center for Biotechnology Information (NCBI) database of expressed sequence tags (dbESTs). All resultant contigs over 200 bp were taken into consideration for the downstream analysis. Because the assembly process may lead to duplicate contigs, CD-HIT-EST was applied with representative sequences at 90% identity to obtain unique contigs (Fu et al., 2012).

In order to estimate contig expression level, we applied the CLC approach to map all fastq format reads back to the contigs and calculated the reads per kilobase of the transcript per million mapped reads (RPKM) values. Because the five samples lacked technical replicates, the non-parametric approach for the identification of differentially expressed genes, NOISeq-sim (Tarazona et al., 2011), was applied to analyze ten independent pair-wise sample comparisons.

### Annotation Pipeline and Data Mining

As query sequences, all assembled contigs were subjected to the BLASTx sequence similarity search against the non-redundant (NR) protein database at NCBI and the Universal Protein resource (UniProt) at UniProt consortium. The *e*-value threshold was set to 1e - 10; the upper limit on the number of subject sequences from databases to show alignment was limited to 20. As to the large BLASTx output, only percent identities over 40% and *e*-values less than 1e - 30 were taken into consideration (Han et al., 2015). After eliminating redundancies, all unique gene identifiers in fasta format were then uploaded to the UniProt ID mapping website for online data processing^2^. By consolidating the returned target list and the UniProtKB ACs obtained from the above-mentioned BLASTx output against the UniProt database, we applied the redundancy-free ACs to annotation using the same online facilities. Out of the huge number of annotation results, we examined the reviewed findings from UniProtKB/Swiss-Prot as well as TrEMBL for data mining (Han et al., 2015). The contigs with GO terms at the protein level were classified. Ultimately, 1,348 enzyme commission (EC) numbers were collected to map pathways against KEGG, and the enzymes related to daidzein biosynthesis were depicted.

### Gene Expression Validation Adopting qRT-PCR

Quantitative real-time reverse transcription polymerase chain reaction was conducted using 96-well plates and StepOnePlus real-time PCR system (Applied biosystems). Three technical replicates were used for each reaction, and a negative control consisting of template without primers was included for each template. Reaction volume was 15 μL and each reaction comprised 7.5 μL of SYBR Select Master mix (2×), 0.15 μL of 10 μM primers (1:1 mix of forward and reverse primers), 1.0 μL of cDNA synthesized using SuperScript VILO cDNA Synthesis Kit (Life technologies), and 6.35 μL of nuclease-free distilled water. Reaction conditions included 10 min incubation at 95°C, then 40 cycles of 95°C for 15 s and 60°C for 1 min, followed by a melt-curve analysis to confirm single PCR product amplification. β-actin was used as the internal control gene (Hong et al., 2010). No amplification was observed in any negative control. Equivalent slopes for target and internal control gene were observed in amplification plots, so the comparative threshold-cycle (C_T_) method was used to calculate relative expression levels as 2^-ΔCt^ where ΔCt = (C_T_ target gene – C_T_ internal control gene), assuming similar PCR efficiencies of target and internal control gene (Schmittgen and Livak, 2008; Gaines et al., 2014).

### HPLC Analysis of Puerarin and Daidzin in Five Tissues of *P. lobata*

One gram of every fresh tissue studied was ground to powder in liquid nitrogen and extracted overnight with 5 ml of acetone at 4°C. Then the extract was centrifuged at 3,500 rpm for 30 min (He et al., 2008, 2011) and the supernatant was dried in ventilator. The residues were resuspended in methanol, and 10 μL of the solution was analyzed by reverse-phase HPLC (HITACHI D7000 system) on a 5-μm C15 column (150 × 4.6 mm, Mightysil). The flow rate was 0.5 mL/min with the mobile phase methanol/water (25:75).

## Results

### Plant RNA Extraction and cDNA Library Preparation

Studies on legumes showed the isoflavones daidzein and genistein were major metabolites in all embryonic organs within the dry seeds. Seedling roots and callus cultures are known to produce daidzein, with the highest daidzein concentration to be found in mature fruits (Graham, 1991; Bouque et al., 1998). We intended collecting information regarding the nature of the genes responsible for the biosynthesis of daidzein and daidzin in *P. lobata*. We extracted total RNA from the leaf, mature root, root vascular cylinder (Root VC), young root and stem of the plant. Five distinct cDNA libraries were established from these five tissue samples. We will refer to the five libraries in the following manner: Library 1 (leaf), Library 2 (mature root), Library 3 (root VC), Library 4 (young root), Library 5 (stem). Wherever applicable, a uniform color scheme will be used to represent the libraries: red (Library 1), purple (Library 2), green (Library 3), blue (Library 4), and yellow (Library 5).

### Illumina Sequencing and *De Novo* Assembly

**FIGURE 1:**
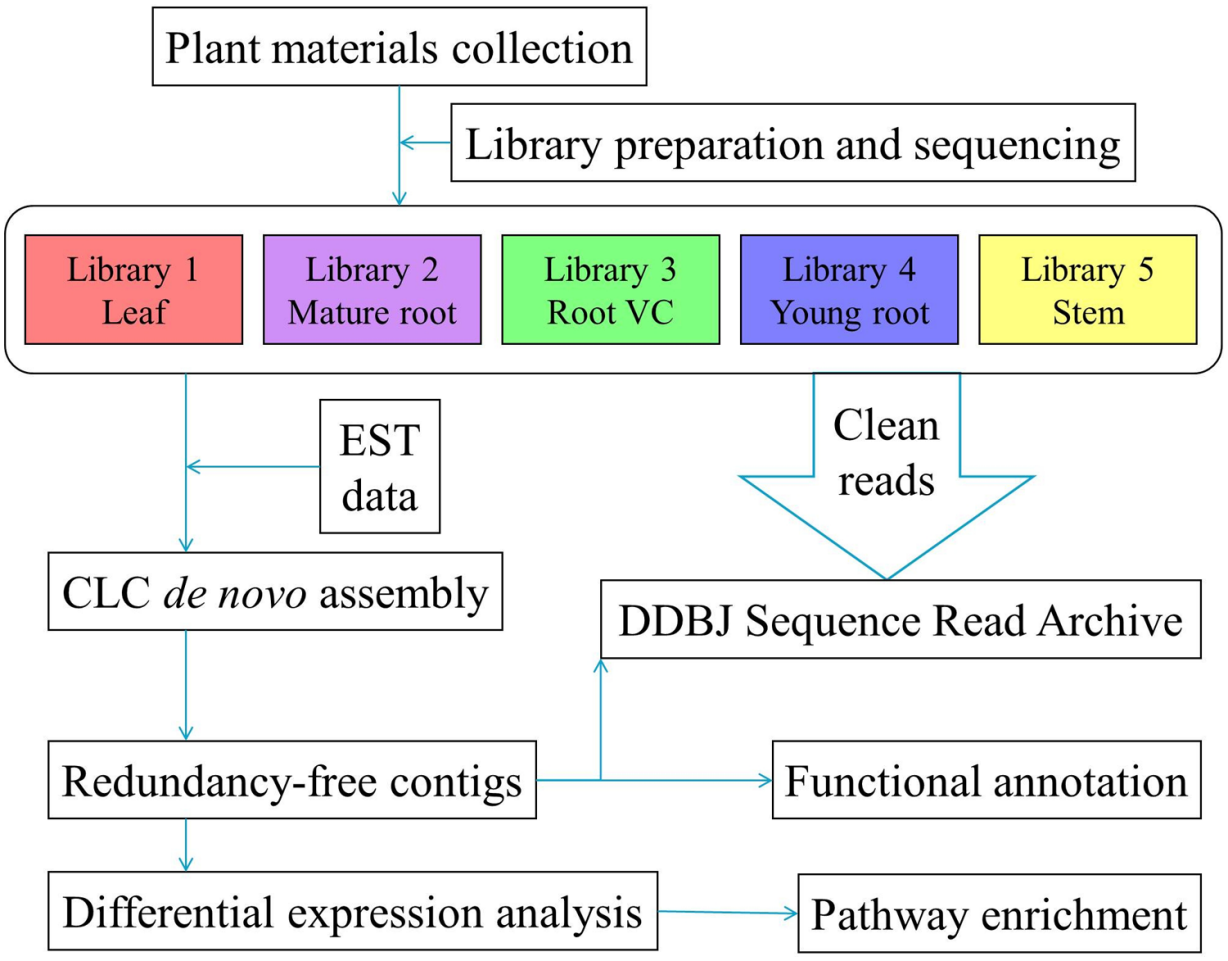
**Summary of the experimental design and analysis pipeline**.

All five libraries were processed using the Illumina HiSeq 1000 platform. Reads of poor quality, empty reads and those with unknown bases were trimmed by CLC software. In order to consolidate the available bio-information to obtain more reliable and thorough findings, we combined the resultant clean reads with *P. lobata* EST sequences obtained from the NCBI database to conduct *de novo* assembly; thus, 83,041 contigs were generated. By adopting CD-HIT-EST with a threshold set to 0.9, duplicates were retrieved and discarded, leaving 81,508 NR contigs for downstream analysis. An overview of the experimental pipeline is shown in **Figure 1**. **Tables 1** and **2** summarize trimming, sequencing, and assembly results.

**Table 1:** Quality control for transcriptomic analysis.

Library	No. of reads (paired)	Average length (bp)	No. of reads after trim (bp)	Average length after trim (bp)
(1) Leaf	18,247,136	101.0	18,189,752	98.8
(2) Mature root	14,090,648	101.0	14,039,985	98.2
(3) Root VC	20,847,896	101.0	20,776,909	98.7
(4) Young root	23,941,990	101.0	23,869,723	98.8
(5) Stem	16,429,912	101.0	16,372,545	98.5

**Table 2:** Overview of *P. lobata* transcriptomic assembly.

Items	Numbers
Total bases	9,197,584,658
Average length of reads (bp)	98.6
No. of reads (6,365 ESTs included)	93,255,279
Average length of contigs (bp)	730
N75; N50; N25 (bp)	488; 1,145; 2,125
No. of contigs over 200 bp	83,041
Non-redundant contigs	81,508


### Guanine–Cytosine (GC) Content Analysis

The reported GC content for unigene sequences in soybean and *Arabidopsis* is 0.43 and 0.44, respectively (Tian et al., 2004; Kawaguchi and Bailey-Serres, 2005). The mean GC content of *P. lobata* transcripts was found to be 39.9% (Supplementary S1). In eukaryotes, average GC content covers the range from ∼20 to 60% (Serres-Giardi et al., 2012). Our values are in the middle of this range, slightly lower than those reported for *Glycine max* (43%) but very close to those reported for *Sophora flavescens* (39.3%) and *Medicago truncatula* (40%) (Tian et al., 2004; Han et al., 2015).

### Transcriptome Information and Differential Accumulation of Transcripts

**FIGURE 2:**
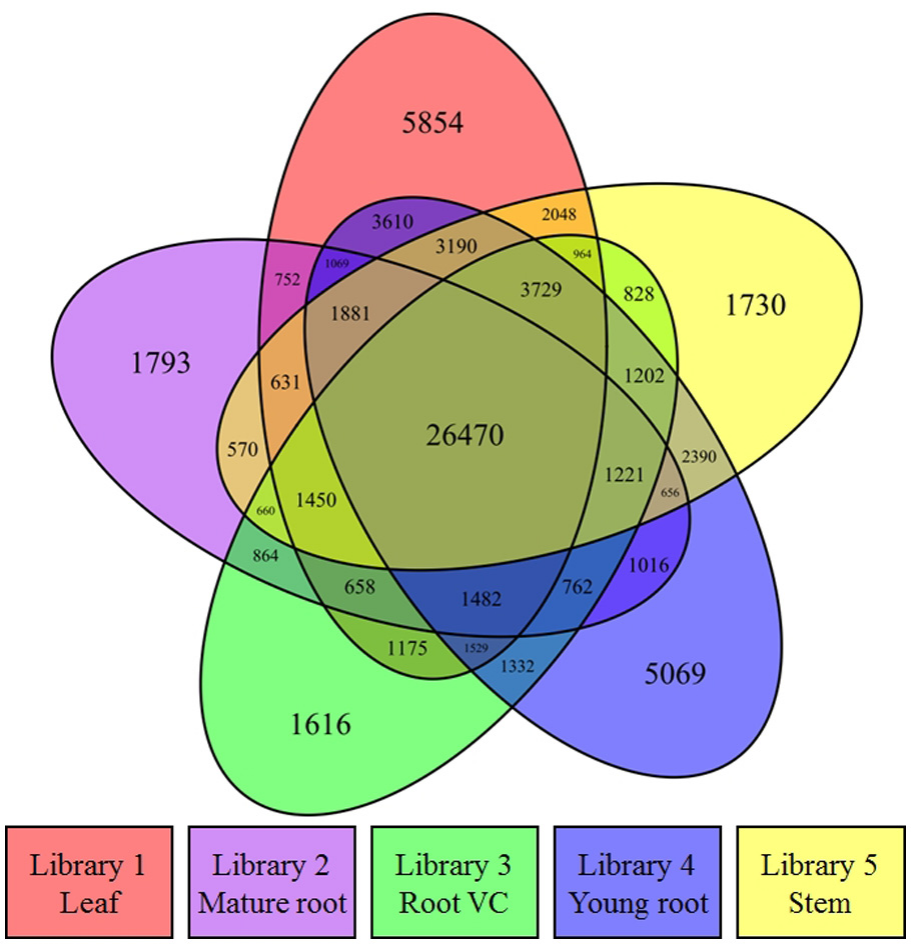
**Transcriptomic expression analysis.** A Venn diagram shows the distribution of transcriptionally active contigs whose RPKM values are greater than 0 in at least one of the libraries.

RPKM calculation was conducted for transcript expression analysis after RNA-seq reads from each library were aligned to all contigs. A Venn diagram was drawn by utilizing the R project in conjunction with the VennDiagram package (Chen and Boutros, 2011) to illustrate the distribution profile of all active contigs (78,201) with RPKM values >0 in at least one of the libraries (**Figure 2**). 33.8% of the active transcripts were expressed across all five libraries, suggesting the homogeneity and high quality of the acquired raw data. Compared to other three tissues, young root and leaf had more exclusively expressed contigs, which demonstrated in spring, such tissues played important and unique physiological roles. RPKM calculation considers gene length variation and the number of total mapped reads, which allows this normalized output to be used directly for the comparison of gene expression. The transcript RPKM values which are greater than 0 in at least one of the libraries are listed in Supplementary S2. To perform differential expression (DE) analysis, 10 pair-wise comparisons for the five libraries were conducted by applying NOISeq-sim, which is a non-parametric approach for the identification of differentially expressed genes from count data or previously normalized count data. By running NOISeq-sim on an R language platform with the given threshold (*q* = 0.9) for selecting differentially expressed features, the resultant number of DE transcripts varied across comparisons (Han et al., 2015). The highest value obtained was 1,408 differences between leaf and mature root transcripts; the lowest value obtained was 297 differences between mature root and root vascular cylinder (Supplementary S3).

### Protein Function Annotations and Gene Ontology Classification

Functional annotations according to sequence similarity are often the initial step in studying the role and biological functions of gene products (Ramilowski et al., 2013). The basic local alignment search tool (BLAST) was utilized to scan nucleotide query sequences against protein databases (NR, UniProt) to identify similar subject sequences. When the threshold *e*-value for BLASTx searches was set to 1e - 10 and the top 20 subject sequences for each query sequence were taken into consideration, we obtained 829,087 subject sequences for all 81,508 query sequences. To obtain reliable results while reducing redundancy, we set stricter requirements for retrieving the candidate genes (Han et al., 2015). With this approach, significant matches were assigned to 30,156 contigs.

**FIGURE 3:**
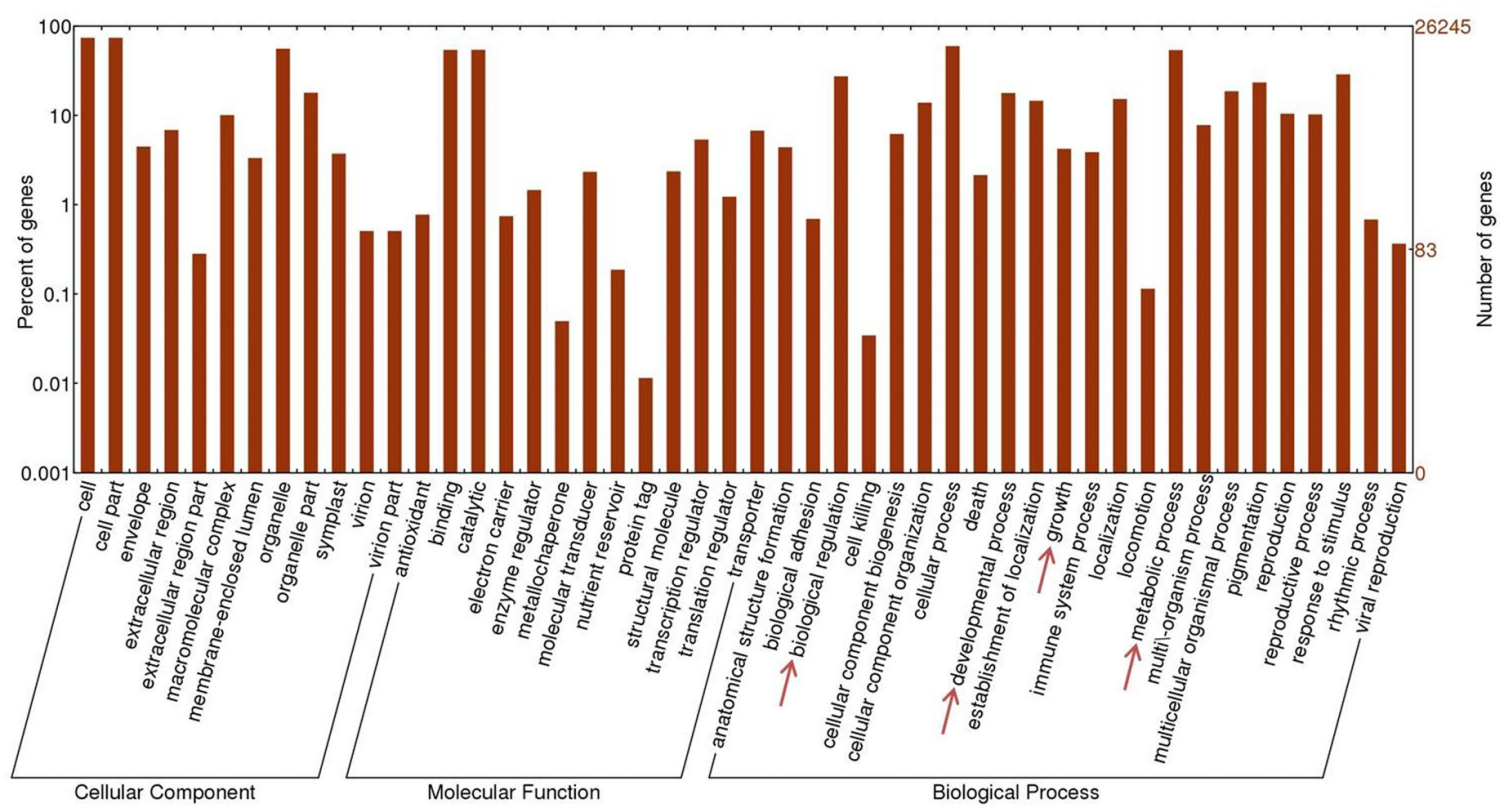
**Gene Ontology annotation for *P. lobata* contigs.** Forty eight subcategories are affiliated to three main domains: molecular function, cellular components and biological processes.

Gene Ontology, comprising domains of biological processes, molecular functions and cellular components, is a useful instrument with which to study the nature of annotated genes (Ashburner et al., 2000). With the help of Web Gene Ontology Annotation Plot (WEGO) software (Ye et al., 2006), 26,245 contigs yielded corresponding GO terms that could be further classified into 48 sub-categories: 12 related to cellular components, 13 to molecular function and 23 to biological processes. **Figure 3** presents the large number of transcripts related to metabolic processes (14,164) and biological regulation (7,194). The contigs assigned to “growth” and “developmental process” under GO biological process may hold the key to the mechanism underlying its rapid and aggressive growth rate. The disadvantage of evaluating gene classification by directly counting the number of GO terms which possess the same or very similar functions is that the expression level of these query sequences varies, which grants distinct weight to the same GO term as it corresponds to different query sequences. With this concern, overrepresented GO terms were identified by Fisher’s exact test. The one-tailed Fisher’s exact *p*-values corresponding to overrepresented categories were calculated according to the counts in 2 × 2 contingency tables. Counts *n*_11_, *n*_12_, *n*_21_, and *n*_22_ in each table stand for: *n*_11_, number of observations of a specific category in the first gene set; *n*_12_, number of other categories in the first gene set; *n*_21_, number of observations of a category in the second gene set; and *n*_22_, number of observations of other categories in the second gene set (Takahashi et al., 2011; Han et al., 2015). *p*-values were corrected utilizing the false discovery rate (FDR) method (Benjamini and Hochberg, 1995) with the threshold of 0.05. For each *P. lobata* library, contigs with RPKM value over 15.0 (the top ∼10% of all transcripts) were regarded as highly expressed genes and extracted respectively. Then the merged 14,364 contigs were used to perform Fisher’s exact test.

As with many woody vines supported by trees or man-made structures, Kudzu may allocate the majority of its biomass to vine elongation and leaf growth. It is regarded as an invasive alien plant in Europe and northern America because its rapid growth rate (up to 30 cm d ^-1^; Lindgren et al., 2013) allows Kudzu to suffocate neighboring plants that are deprived of sunlight. The overrepresented GO terms (Supplementary S4, GO terms with *p* < 1E - 30 are listed) suggest that highly activated biological processes such as cell division (GO: 0051301), cell growth (GO: 0016049), root hair elongation (GO: 0048767), response to cold (GO: 0009409) and response to salt stress (GO: 0009651) could play an essential role in its aggressive development.

### KEGG Pathway Retrieval

The Kyoto Encyclopedia of Genes and Genomes (KEGG) provides a robust instrument for biological pathway assignment as well as the functional annotation of gene products (Han et al., 2015). From ID mapping results, we obtained 1,348 unique enzymes corresponding to 16,380 contigs and subsequently retrieved pathways using KEGG. The EC numbers were assigned to 152 biological pathways with more than half of the enzymes (697) involved in metabolic pathways. Regarding the significant capacity of leguminous plants to accumulate functional flavonoids, 19 flavonoid biosynthetic and 14 isoflavonoid biosynthetic enzymes are presented in Supplementary S5.

### Genes Involved in Isoflavonoid Biosynthesis in *P. lobata*

Flavonoids are a group of polyphenolic compounds distributed widely throughout the plant kingdom. These compounds modulate the activity of enzymes to benefit the entire organism. As an important subgroup of flavonoids, isoflavonoids are mainly produced in legumes and affect oxidative stress markers, immune function and adipogenesis (Miadokova, 2009; Han et al., 2015).

**FIGURE 4:**
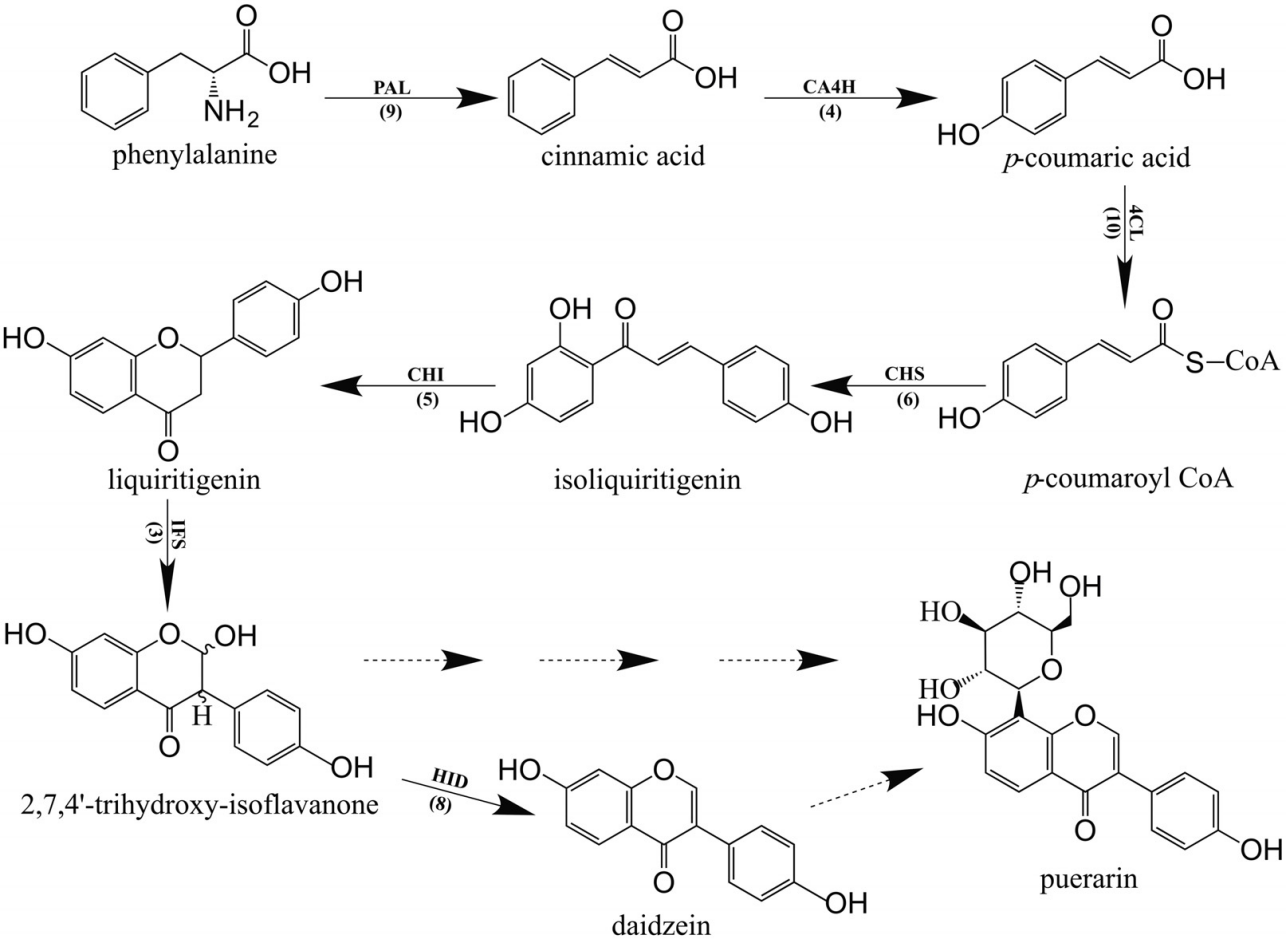
**Proposed daidzein biosynthesis pathway in *P. lobata*.** Every enzyme is followed by the number of corresponding contigs in parentheses. PAL, Phenylalanine ammonia-lyase, EC 4.3.1.24; CA4H, *Trans*-cinnamate 4-monooxygenase, EC 1.14.13.11; 4CL, 4-coumarate-CoA ligase, EC 6.2.1.12; CHS, 6′-deoxychalcone synthase, EC 2.3.1.170; CHI, Chalcone isomerase, EC 5.5.1.6; IFS, 2-hydroxyisoflavanone synthase, EC 1.14.13.136; HID, 2-hydroxyisoflavanone dehydratase, EC 4.2.1.105. Solid arrows show the pathways identified by the data obtained while the dotted arrows show unsolved steps.

**FIGURE 5:**
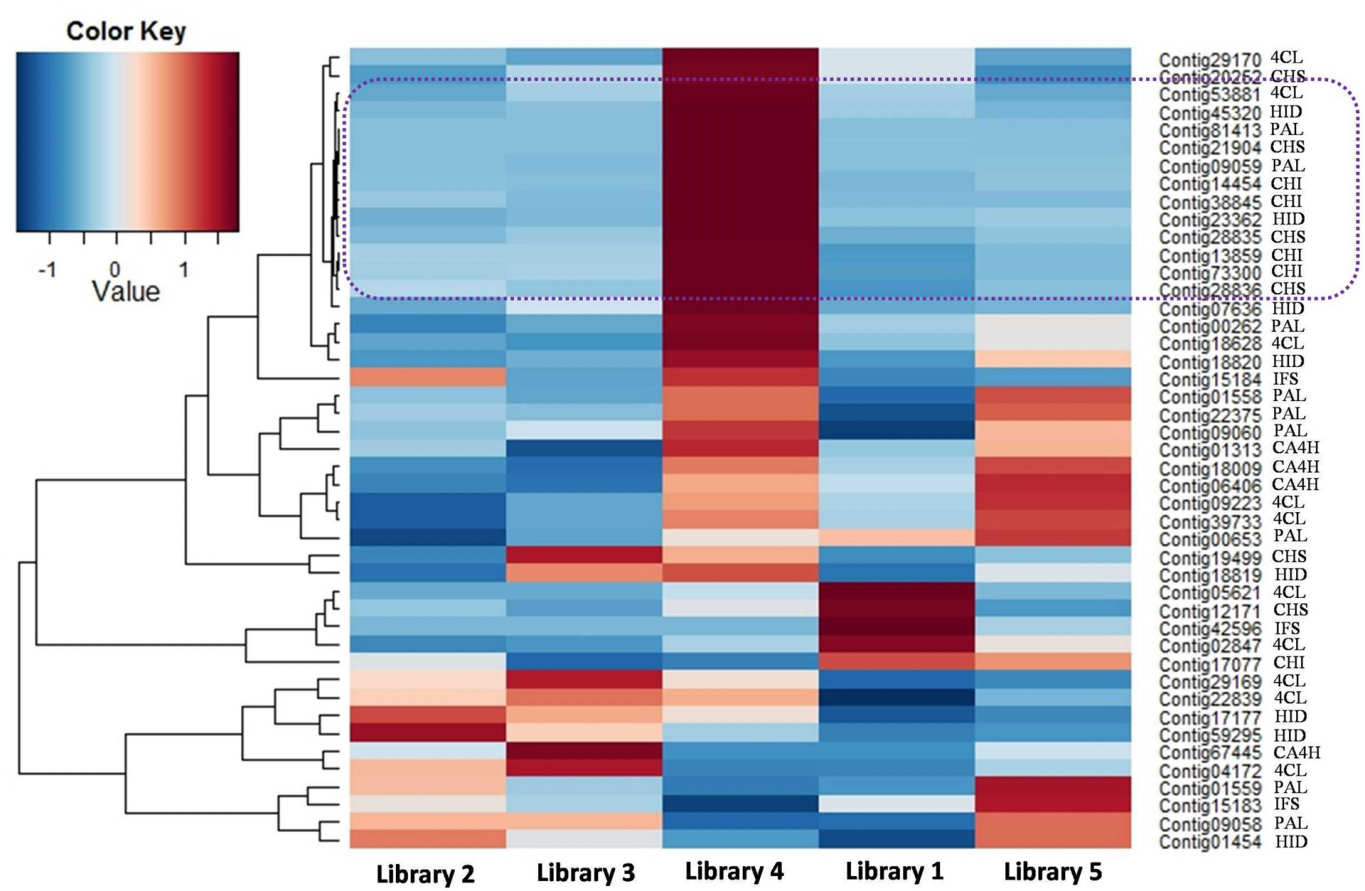
**Heatmap showing the expression profile for 45 contigs related to daidzein biosynthesis.** Next to each contig name, the enzyme abbreviation is presented.

In the phenylpropanoid pathway, the synthesis of flavonoids is initialized by transforming phenylalanine into *p*-coumaroyl-CoA. To initiate flavonoid biosynthesis, chalcone synthase catalyzes the formation of chalcone scaffolds, from which all flavonoids derive (Falcone Ferreyra et al., 2012; Saito et al., 2013; Han et al., 2015). Based on our functional annotation findings, 45 contigs were predicted to represent seven enzymes critical to the biosynthesis of daidzein, which may be necessary to make the puerarin found in *P. lobata*. The number of contigs corresponding to each enzyme and the biosynthetic pathway are presented in **Figure 4**. **Figure 5** shows the expression profile for the 45 contigs corresponding to seven daidzein-biosynthesis-related enzymes across 5 libraries. From the heatmap, majority of the contigs had strong expression in library 4 (young root) and high correlation could be found among phenylalanine ammonia-lyase (PAL), 4-coumarate-CoA ligase (4CL), 6′-deoxychalcone synthase (CHS), chalcone isomerase (CHI) and 2-hydroxyisoflavanone dehydratase (HID), which gave light to the biosynthetic pathway of flavonoids in *P. lobata*. A previous study (Chen et al., 2001) showed that concentrations of puerarin and daidzein were up to threefold higher in the roots as compared to the veins of *P. lobata*. In our study, biosynthesis of the chalcone scaffold showed that the expression of downstream enzymes exhibits a clear pattern that provides evidence into the organ-specific biosynthesis of daidzein (Supplementary S6). In *P. lobata*, early steps for the biosynthesis of daidzein and even puerarin might take place mainly in young root. After transportation of the required precursors to other parts of the plant or along with organ growth, the expression of related enzymes increases in mature root. Finally, the high expression of 2-hydroxyisoflavanone dehydratase in stem and mature root may account for the accumulation of daidzein and puerarin in certain plant organs.

**Figure 4** demonstrates 45 contigs involved in the isoflavonoid biosynthetic pathway. We carried out to validate the genuine biological expression profile of such contigs, focusing on the genes from chalcone synthase (EC 2.3.1.170) to 2-hydroxyisoflavanone dehydratase (EC 4.2.1.105) since these are crucial factors leading to the accumulation of isoflavonoids. According to Supplementary S6, several contigs can be aligned to each of the four enzymes. Therefore, we set the following criteria for selecting the appropriate candidate contigs in qRT-PCR experiment: firstly, the contig itself should consist of long fragment; secondly, the expression judging by RPKM values across the five tissues was relatively high; thirdly, the identity between the candidate contig and target enzyme should be high. Contig 21904, 14454, 15184, and 01454, corresponding to chalcone synthase, chalcone isomerase, 2-hydroxyisoflavanone synthase and 2-hydroxyisoflavanone dehydratase, respectively, were chosen to perform qRT-PCR. For CHS and CHI, **Table 3** shows similar pattern of gene expression profile between deep transcriptomic data and qRT-PCR result. Regarding chalcone synthase, its DE between young root and leaf was detected by NOISeq-sim and the ratio of 2^-ΔCt^ was also the highest one, 36. However, although the trend that the expression of 2-hydroxyisoflavanone synthase (IFS) and HID in library 1 was always lower compared to other libraries was verified by both approaches, the ratio varied. For example, RPKM value suggested 20.1-fold elevated expression in library 2 while qRT-PCR granted only 2.90-fold. We collected the samples for sequencing in 2012 but the materials used for qRT-PCR were obtained in 2014 from the same location, though. The slightly changed sampling conditions may result in the variations in validation experiment. The primers designed for qRT-PCR are listed in Supplementary S7.

**Table 3:** Validation of differentially expressed genes related to isoflavonoid biosynthesis.

Contig	Annotation	Fold change : RNA-Seq and qRT-PCR validation							
		Lib 2/Lib 1		Lib 4/Lib 1		Lib 3/Lib 1		Lib 5/Lib 1	
		RPKM	2^--ΔCt^	RPKM	2^--ΔCt^	RPKM	2^--ΔCt^	RPKM	2^--ΔCt^
21904	CHS	0.49	1.87	48∗	36	0.76	0.97	0.47	0.60
14454	CHI	1.20	0.72	9.0	14.6	1.12	0.63	1.29	2.07
15184	IFS	20.1	2.90	25.1	6.84	3.31	2.45	2.69	2.14
01454	HID	5.87	7.86	2.27	12.3	4.04	8.51	6.07	25.1

*Fold change of RPKM and 2^-ΔCt^ between Lib 1 (Library 1) and other Libraries, respectively. ∗differently expressed gene detected by NOISeq-sim. CHS chalcone synthase, CHI Chalcone isomerase, IFS 2-hydroxyisoflavanone synthase, HID 2-hydroxyisoflavanone dehydratase*.

## Discussion

From the expression pattern of isoflavonoid biosynthesis in Kudzu, transportation of certain precursors into other parts of the plant for downstream reaction may be required (refer to genes involved in isoflavonoid biosynthesis in *P. lobata*). By searching the annotation data, 41 expressed ABC transporters were retrieved. Regarding IFS and HID, suggested by the changes of expression level in different tissues, transportation of the intermediates may occur. Supplementary S8 lists 41 ABC transporters found in Kudzu dataset along with Pearson correlation coefficients to contig 15184 which is a putative IFS and contig 01454, an annotated HID.

A recent study provided fairly constructive insights into the biosynthetic pathway of puerarin and contributed more than 6,365 ESTs (He et al., 2011). We integrated the publically available ESTs into our raw reads from five different tissues of Kudzu and then performed *de novo* assembly altogether. This enabled us to utilize related information and the assembled contigs showed identical or highly similar transcripts with the ESTs regarding glucosyltransferase.

Many *C*-glucosyltransferases have been identified in bacteria, insects and plants, especially in cereals. The elucidated mechanism for *C*-glycosylation of flavonoids proved 2-hydroxylation of flavanones was the appropriate premise for the catalytic reaction to proceed. Likewise, in studying the biosynthesis of puerarin, 2-hydroxylation of isoflavanone (2,7,4′-trihydroxy-isoflavanone) should be considered as a possible substrate for its formation when daidzein as a direct putative precursor meets with obstacles (**Figure 4**). With the formation of trihydroxy-isoflavone 8-*C* glycoside catalyzed by suitable UDP-dependent glucosyltransferases, the glycoside may be subjected to dehydration reaction, resulting in puerarin. Forty nine contigs were annotated as glucosyltransferase in our dataset, if the target glucosyltransferase utilizes either one of the above-mentioned precursors, the correlation with the enzyme directly producing 2,7,4′-trihydroxy-isoflavanone or daidzein would be significant. **Table 4** lists the annotated glucosyltransferases along with Pearson correlation coefficients to HID and IFS.

**Table 4:** Putative glucosyltransferases with Pearson correlation coefficients to HID and IFS.

Contig_ID	Coefficient_to_HID	Coefficient_to_IFS	Annotation
Contig02990	0.37	0	Sucrose-UDP glucosyltransferase
Contig03131	-0.4	0.74	Anthocyanidin 3-*O*-glucosyltransferase
Contig03133	-0.45	0.48	Anthocyanidin 3-*O*-glucosyltransferase
Contig05593	-0.59	0.53	Glycoprotein glucosyltransferase
Contig09646	-0.68	0.31	Cytokinin-*O*-glucosyltransferase
Contig10691	-0.59	0.45	glycoprotein glucosyltransferase
Contig11620	0.79	0.1	Putative glucosyltransferase
Contig11621	-0.69	-0.4	Putative glucosyltransferase
Contig11622	-0.84	-0.39	Putative glucosyltransferase
Contig12257	-0.66	-0.54	Cytokinin-*O*-glucosyltransferase
Contig14425	-0.77	0.47	Putative UDP-glucosyltransferase
Contig15137	-0.6	0.54	glycoprotein glucosyltransferase
Contig15603	-0.72	-0.47	Isoflavonoid glucosyltransferase
Contig20530	-0.78	-0.3	Sterol 3-β-glucosyltransferase
Contig22923	-0.07	0.2	Sterol 3-β-glucosyltransferase
Contig22924	-0.94	0.04	Sterol 3-β-glucosyltransferase
Contig23483	-0.4	0.73	Zeatin *O*-glucosyltransferase
Contig23956	-0.35	0.79	Isoflavonoid glucosyltransferase
Contig25035	-0.19	0.94	Putative glucosyltransferase
Contig28030	-0.29	-0.39	flavonoid 3-*O*-glucosyltransferase
Contig28462	-0.03	0.46	Hydroquinone glucosyltransferase
Contig29838	-0.47	0.22	Isoflavone 7-*O*-glucosyltransferase 1
Contig29839	0.15	-0.43	Isoflavone 7-*O*-glucosyltransferase 1
Contig31158	0.34	-0.12	Cytokinin-*O*-glucosyltransferase
Contig32270	-0.39	0.71	Anthocyanidin 3-*O*-glucosyltransferase
Contig32277	-0.13	0.58	Hydroquinone glucosyltransferase
Contig40732	-0.67	0.07	Sucrose-UDP glucosyltransferase
Contig42441	-0.9	-0.18	Cytokinin-*O*-glucosyltransferase
Contig43033	0.27	-0.7	Isoflavone 7-*O*-glucosyltransferase 1
Contig44304	-0.15	0.59	Isoflavone 7-*O*-glucosyltransferase 1
Contig45936	-0.04	-0.98	Isoflavone 7-*O*-glucosyltransferase 1
Contig46490	0.34	-0.55	Anthocyanidin 3-*O*-glucosyltransferase
Contig47890	-0.28	0.75	Sterol 3-β-glucosyltransferase
Contig51492	-0.41	0.7	UDP-glucosyltransferase
Contig52569	-0.22	0.64	Sterol 3-β-glucosyltransferase
Contig65011	-0.4	0.68	UDP-glucosyltransferase
Contig73434	0.56	-0.38	Limonoid UDP-glucosyltransferase
Contig74493	0.1	0.21	Anthocyanidin 3-*O*-glucosyltransferase
Contig77022	-0.35	0.47	Anthocyanidin 3-*O*-glucosyltransferase
Contig79587	-0.4	0.73	Cytokinin-*O*-glucosyltransferase
Contig79864	-0.4	0.73	Cytokinin-*O*-glucosyltransferase
Contig03787	0.47	-0.6	Glucosyltransferase
Contig06374	-0.83	0.07	Glucosyltransferase-13 (Fragment)
Contig11423	-0.71	-0.49	Glucosyltransferase
Contig14082	-0.58	-0.42	Glucosyltransferase-2
Contig14083	-0.6	-0.35	Glucosyltransferase-12
Contig14085	0.3	0.89	Glucosyltransferase-2
Contig24631	0.04	0.67	Glucosyltransferase-5
Contig28995	-0.33	0.73	Glucosyltransferase-12

Kudzu root, which is the main part prescribed in oriental medicines in treating various diseases, produce predominantly isoflavone *C-* and *O-* glucosides. We collected five tissues from which the deep transcriptomic data were generated and studied puerarin and daidzin profile using HPLC. Both puerarin and daidzin are highly accumulated in mature root and root vascular cylinder and the concentration of puerarin is higher than that of daidzin (**Table 5**, Supplementary S9). Although several genes related to isoflavonoid biosynthesis are highly expressed in young root, the concentration of the two compounds is low in young root compared with that in mature root. This may be due to the essential enzymes for the production of puerarin actively expressed in the young and mature roots but the accumulation of puerarin does not reach to the maximum yet in the young root. Deep transcriptomic data obtained in this study may provide the key to this question. In relatively young stem which was used in this study and leaf, daidzin was not detectable.

**Table 5:** Determination of puerarin and daidzin in fresh plant samples.

	Leaf	Stem	Mature root	Young root	Root VC
Puerarin	N.D.^a^	1.473 ± 0.007^b^	4.024 ± 0.005	0.231 ± 0.002	3.327 ± 0.005
Daidzin	N.D.	N.D.	0.668 ± 0.004	0.156 ± 0.002	0.994 ± 0.008

*^a^N.D. = Not detected. ^b^Means ± SD (mg/g fresh weight, n = 3)*.

RNA-Seq analysis is cost effective and the most efficient approach currently available to manage high-throughput data. By consolidating data information obtained from five *P. lobata* libraries, we analyzed the DE profile and mapped biosynthetic pathways against KEGG using enzyme ACs. Evaluating overrepresented GO terms by considering the RPKM values of the corresponding contigs provided a more accurate representation of the data. By qRT-PCR and HPLC, both gene expression validation and metabolite analysis were performed. The deep transcriptomic data we present here may facilitate future research on this promising plant.

## Author Contributions

KS and MY designed the research framework. MN conducted total RNA extraction. HS and DS performed deep-transcriptome sequencing. RH, HT conducted the data interpretation and laboratory analysis. RH, KS and NY contributed to the manuscript preparation.

## Conflict of Interest Statement

The authors declare that the research was conducted in the absence of any commercial or financial relationships that could be construed as a potential conflict of interest.

## Footnotes

1 https://github.com/rongchunhan/Pueraria_lobata

2 http://www.uniprot.org
