# Supplementary material for: Transcriptomic landscape of Pueraria lobata demonstrates potential for phytochemical study
Source: Front Plant Sci. 2015 Jun 22;6:426. doi: 10.3389/fpls.2015.00426 (PMC4476104; doi:10.3389/fpls.2015.00426)
Supplement: Supplementary file 1 [file Data_Sheet_1.DOCX]

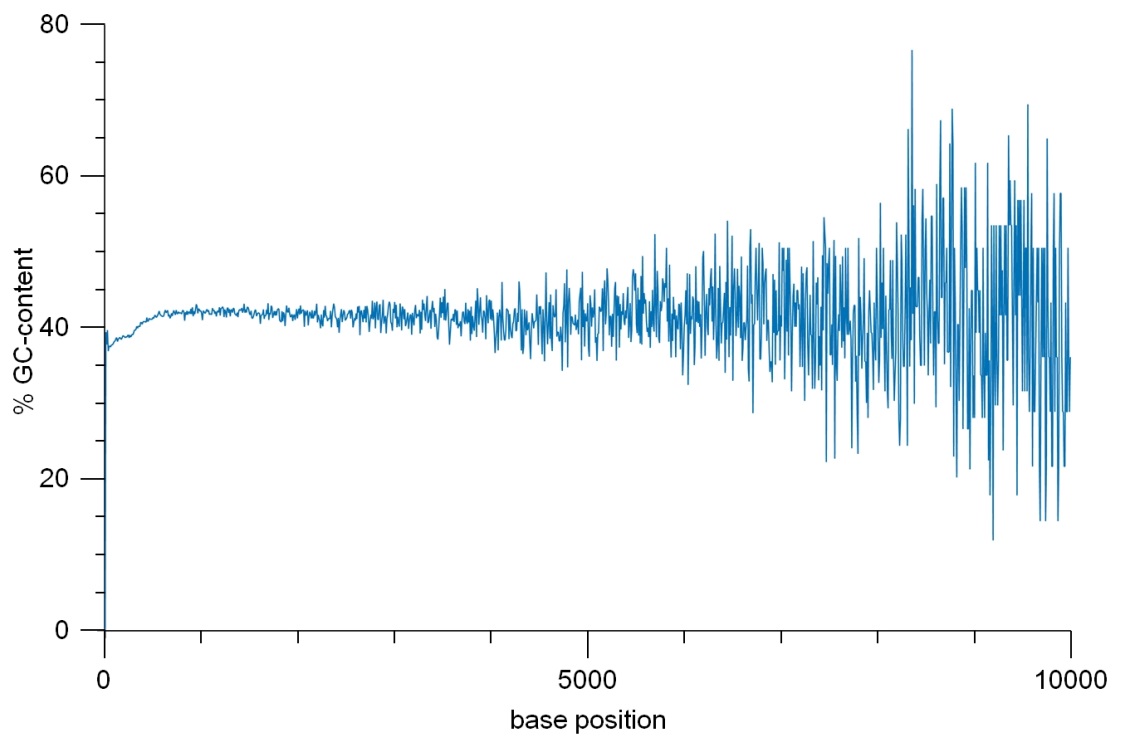


**Supplementary 1** GC content at different base positions for all *P. lobata* contigs. The length of the contigs varies from 200 to 15,631 bp. However, the GC content presented here does not calculate contigs information for more than 10,000 bp.
