# Supplementary material for: Transcriptomic landscape of Pueraria lobata demonstrates potential for phytochemical study
Source: Front Plant Sci. 2015 Jun 22;6:426. doi: 10.3389/fpls.2015.00426 (PMC4476104; doi:10.3389/fpls.2015.00426)
Supplement: Supplementary file 3 [file Data_Sheet_3.DOCX]

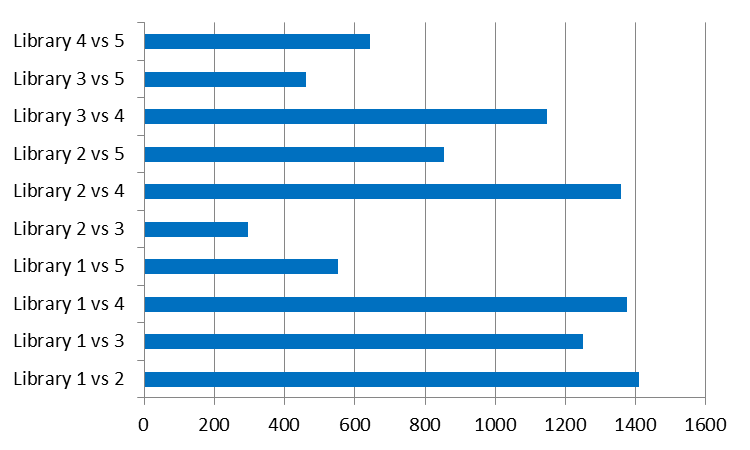


**Supplementary 3** Number of differentially expressed transcripts determined by NOISeq-sim for pairwise comparisons among the 5 libraries.
