# Supplementary material for: Transcriptomic landscape of Pueraria lobata demonstrates potential for phytochemical study
Source: Front Plant Sci. 2015 Jun 22;6:426. doi: 10.3389/fpls.2015.00426 (PMC4476104; doi:10.3389/fpls.2015.00426)
Supplement: Supplementary file 4 [file Data_Sheet_4.DOCX]

**Supplementary 4** Overrepresented GO terms resulted from Fisher’s exact test.

| **Probability** | **GO term** | **Gene_ontology_name** | **n11 n12 n21 n22** |
| --- | --- | --- | --- |
| 0.00E+00 | GO:0005737 | cytoplasm | 1311 1152 13053 65992 |
| 0.00E+00 | GO:0009507 | chloroplast | 1561 1376 12803 65768 |
| 0.00E+00 | GO:0005829 | cytosol | 1225 462 13139 66682 |
| 0.00E+00 | GO:0016021 | integral component of membrane | 1700 2082 12664 65062 |
| 0.00E+00 | GO:0005634 | nucleus | 3346 3560 11018 63584 |
| 4.61E-239 | GO:0005794 | Golgi apparatus | 649 356 13715 66788 |
| 8.24E-233 | GO:0009570 | chloroplast stroma | 514 179 13850 66965 |
| 1.04E-222 | GO:0009506 | chloroplast stroma | 621 357 13743 66787 |
| 3.01E-219 | GO:0046872 | metal ion binding | 796 683 13568 66461 |
| 1.88E-200 | GO:0005524 | ATP binding | 1663 3003 12701 64141 |
| 4.52E-195 | GO:0046686 | response to cadmium ion | 387 98 13977 67046 |
| 1.13E-178 | GO:0016020 | membrane | 732 723 13632 66421 |
| 7.99E-177 | GO:0009651 | response to salt stress | 429 186 13935 66958 |
| 3.44E-165 | GO:0005774 | vacuolar membrane | 385 152 13979 66992 |
| 1.47E-160 | GO:0009535 | chloroplast thylakoid membrane | 306 67 14058 67077 |
| 2.63E-159 | GO:0009941 | chloroplast envelope | 382 161 13982 66983 |
| 1.31E-156 | GO:0005739 | mitochondrion | 971 1415 13393 65729 |
| 7.31E-143 | GO:0005576 | extracellular region | 679 781 13685 66363 |
| 3.03E-132 | GO:0005783 | endoplasmic reticulum | 371 213 13993 66931 |
| 1.54E-118 | GO:0006351 | transcription, DNA-templated | 532 575 13832 66569 |
| 1.64E-115 | GO:0003700 | sequence-specific DNA binding transcription factor activity | 594 733 13770 66411 |
| 3.31E-111 | GO:0000166 | nucleotide binding | 339 223 14025 66921 |
| 5.76E-108 | GO:0003677 | DNA binding | 803 1324 13561 65820 |
| 1.26E-104 | GO:0005730 | nucleolus | 227 74 14137 67070 |
| 2.64E-103 | GO:0009409 | response to cold | 251 108 14113 67036 |
| 6.59E-103 | GO:0006412 | translation | 288 164 14076 66980 |
| 8.16E-102 | GO:0006096 | glycolytic process | 215 65 14149 67079 |
| 9.65E-100 | GO:0008270 | zinc ion binding | 730 1189 13634 65955 |
| 5.94E-99 | GO:0003735 | structural constituent of ribosome | 284 169 14080 66975 |
| 1.21E-98 | GO:0006364 | rRNA processing | 210 65 14154 67079 |
| 1.85E-98 | GO:0006098 | pentose-phosphate shunt | 184 37 14180 67107 |
| 2.33E-98 | GO:0005840 | ribosome | 276 158 14088 66986 |
| 3.00E-98 | GO:0019288 | isopentenyl diphosphate biosynthetic process, methylerythritol 4-phosphate pathway | 211 67 14153 67077 |
| 1.73E-94 | GO:0005773 | vacuole | 257 139 14107 67005 |
| 3.69E-94 | GO:0009611 | response to wounding | 224 92 14140 67052 |
| 4.11E-93 | GO:0005802 | trans-Golgi network | 223 93 14141 67051 |
| 3.90E-92 | GO:0006355 | regulation of transcription, DNA-templated | 469 572 13895 66572 |
| 1.84E-89 | GO:0010200 | response to chitin | 250 142 14114 67002 |
| 1.85E-88 | GO:0007030 | Golgi organization | 171 39 14193 67105 |
| 7.18E-88 | GO:0016787 | hydrolase activity | 374 381 13990 66763 |
| 2.65E-87 | GO:0003824 | catalytic activity | 359 352 14005 66792 |
| 8.27E-86 | GO:0005768 | endosome | 228 118 14136 67026 |
| 2.69E-84 | GO:0016192 | vesicle-mediated transport | 188 66 14176 67078 |
| 3.08E-84 | GO:0005618 | cell wall | 278 206 14086 66938 |
| 2.06E-83 | GO:0048046 | apoplast | 244 150 14120 66994 |
| 2.48E-79 | GO:0003723 | RNA binding | 469 650 13895 66494 |
| 4.64E-79 | GO:0019344 | cysteine biosynthetic process | 178 64 14186 67080 |
| 6.25E-75 | GO:0006511 | ubiquitin-dependent protein catabolic process | 184 81 14180 67063 |
| 2.17E-74 | GO:0042742 | defense response to bacterium | 216 131 14148 67013 |
| 1.21E-73 | GO:0051788 | response to misfolded protein | 140 30 14224 67114 |
| 5.34E-73 | GO:0009737 | response to abscisic acid | 230 159 14134 66985 |
| 1.03E-72 | GO:0010027 | thylakoid membrane organization | 170 67 14194 67077 |
| 3.46E-72 | GO:0048193 | Golgi vesicle transport | 159 54 14205 67090 |
| 5.07E-72 | GO:0045893 | positive regulation of transcription, DNA-templated | 271 239 14093 66905 |
| 5.54E-72 | GO:0006094 | gluconeogenesis | 145 38 14219 67106 |
| 3.20E-71 | GO:0010363 | regulation of plant-type hypersensitive response | 226 158 14138 66986 |
| 5.31E-71 | GO:0006612 | protein targeting to membrane | 225 157 14139 66987 |
| 7.15E-71 | GO:0015031 | protein transport | 186 94 14178 67050 |
| 9.86E-70 | GO:0009853 | photorespiration | 127 23 14237 67121 |
| 3.32E-69 | GO:0009414 | response to water deprivation | 196 114 14168 67030 |
| 1.44E-68 | GO:0009793 | embryo development ending in seed dormancy | 278 268 14086 66876 |
| 2.28E-68 | GO:0080129 | proteasome core complex assembly | 111 11 14253 67133 |
| 8.76E-66 | GO:0010207 | photosystem II assembly | 130 32 14234 67112 |
| 8.08E-65 | GO:0006886 | intracellular protein transport | 164 77 14200 67067 |
| 8.77E-65 | GO:0006457 | protein folding | 204 141 14160 67003 |
| 7.03E-64 | GO:0006508 | proteolysis | 362 484 14002 66660 |
| 5.43E-63 | GO:0016567 | protein ubiquitination | 263 262 14101 66882 |
| 1.79E-61 | GO:0019252 | starch biosynthetic process | 152 68 14212 67076 |
| 3.56E-61 | GO:0005525 | GTP binding | 206 157 14158 66987 |
| 4.42E-61 | GO:0009733 | response to auxin | 183 117 14181 67027 |
| 7.84E-59 | GO:0005777 | peroxisome | 151 73 14213 67071 |
| 4.61E-58 | GO:0050832 | defense response to fungus | 190 139 14174 67005 |
| 7.22E-58 | GO:0009744 | response to sucrose | 138 57 14226 67087 |
| 2.71E-57 | GO:0005507 | copper ion binding | 173 112 14191 67032 |
| 3.49E-57 | GO:0009867 | jasmonic acid mediated signaling pathway | 171 109 14193 67035 |
| 9.04E-57 | GO:0000139 | Golgi membrane | 156 86 14208 67058 |
| 1.18E-56 | GO:0006635 | fatty acid beta-oxidation | 141 64 14223 67080 |
| 1.39E-56 | GO:0009738 | abscisic acid-activated signaling pathway | 174 116 14190 67028 |
| 5.56E-55 | GO:0009902 | chloroplast relocation | 100 18 14264 67126 |
| 9.71E-55 | GO:0015995 | chlorophyll biosynthetic process | 113 32 14251 67112 |
| 3.25E-54 | GO:0004842 | ubiquitin-protein transferase activity | 183 140 14181 67004 |
| 3.67E-54 | GO:0019761 | glucosinolate biosynthetic process | 118 39 14246 67105 |
| 5.59E-54 | GO:0009505 | plant-type cell wall | 165 109 14199 67035 |
| 9.20E-54 | GO:0006979 | response to oxidative stress | 164 108 14200 67036 |
| 3.82E-53 | GO:0004674 | protein serine/threonine kinase activity | 593 1228 13771 65916 |
| 1.67E-52 | GO:0016491 | oxidoreductase activity | 262 313 14102 66831 |
| 1.28E-51 | GO:0008152 | metabolic process | 218 220 14146 66924 |
| 1.68E-51 | GO:0046777 | protein autophosphorylation | 149 90 14215 67054 |
| 3.62E-51 | GO:0006623 | protein targeting to vacuole | 114 40 14250 67104 |
| 1.27E-50 | GO:0048767 | root hair elongation | 148 91 14216 67053 |
| 2.35E-50 | GO:0006833 | water transport | 99 24 14265 67120 |
| 2.97E-50 | GO:0030968 | endoplasmic reticulum unfolded protein response | 136 73 14228 67071 |
| 2.79E-49 | GO:0005509 | calcium ion binding | 198 189 14166 66955 |
| 3.92E-49 | GO:0031348 | negative regulation of defense response | 162 120 14202 67024 |
| 4.78E-49 | GO:0016126 | sterol biosynthetic process | 121 54 14243 67090 |
| 6.02E-49 | GO:0019013 | viral nucleocapsid | 90 17 14274 67127 |
| 8.26E-49 | GO:0034976 | response to endoplasmic reticulum stress | 122 56 14242 67088 |
| 1.04E-48 | GO:0016117 | carotenoid biosynthetic process | 106 35 14258 67109 |
| 1.21E-48 | GO:0000023 | maltose metabolic process | 121 55 14243 67089 |
| 6.05E-48 | GO:0001510 | RNA methylation | 98 27 14266 67117 |
| 1.71E-47 | GO:0005789 | endoplasmic reticulum membrane | 156 115 14208 67029 |
| 2.20E-46 | GO:0015979 | photosynthesis | 110 45 14254 67099 |
| 3.68E-45 | GO:0009407 | toxin catabolic process | 113 52 14251 67092 |
| 9.78E-45 | GO:0009750 | response to fructose | 108 46 14256 67098 |
| 2.95E-44 | GO:0042744 | hydrogen peroxide catabolic process | 105 43 14259 67101 |
| 3.45E-44 | GO:0010228 | vegetative to reproductive phase transition of meristem | 184 183 14180 66961 |
| 1.24E-43 | GO:0009627 | systemic acquired resistance | 121 68 14243 67076 |
| 1.31E-43 | GO:0009862 | systemic acquired resistance, salicylic acid mediated signaling pathway | 129 81 14235 67063 |
| 2.37E-43 | GO:0009220 | pyrimidine ribonucleotide biosynthetic process | 102 41 14262 67103 |
| 6.58E-43 | GO:0035304 | regulation of protein dephosphorylation | 99 38 14265 67106 |
| 6.58E-43 | GO:0030529 | ribonucleoprotein complex | 99 38 14265 67106 |
| 8.87E-43 | GO:0000165 | MAPK cascade | 209 245 14155 66899 |
| 1.42E-42 | GO:0043565 | sequence-specific DNA binding | 221 274 14143 66870 |
| 2.51E-42 | GO:0006007 | glucose catabolic process | 76 13 14288 67131 |
| 2.51E-42 | GO:0005975 | carbohydrate metabolic process | 246 336 14118 66808 |
| 5.90E-42 | GO:0009536 | plastid | 104 47 14260 67097 |
| 1.00E-41 | GO:0006972 | hyperosmotic response | 83 21 14281 67123 |
| 1.40E-41 | GO:0009697 | salicylic acid biosynthetic process | 124 79 14240 67065 |
| 1.40E-41 | GO:0009723 | response to ethylene | 124 79 14240 67065 |
| 1.76E-41 | GO:0009644 | response to high light intensity | 125 81 14239 67063 |
| 6.58E-41 | GO:0009658 | chloroplast organization | 109 57 14255 67087 |
| 8.30E-41 | GO:0009695 | jasmonic acid biosynthetic process | 97 40 14267 67104 |
| 1.59E-40 | GO:0009640 | photomorphogenesis | 151 132 14213 67012 |
| 5.76E-40 | GO:0032440 | 2-alkenal reductase [NAD(P)] activity | 257 380 14107 66764 |
| 5.86E-40 | GO:0006499 | N-terminal protein myristoylation | 104 52 14260 67092 |
| 1.46E-39 | GO:0005622 | intracellular | 150 134 14214 67010 |
| 1.50E-39 | GO:0009408 | response to heat | 137 109 14227 67035 |
| 3.67E-39 | GO:0003743 | translation initiation factor activity | 111 65 14253 67079 |
| 1.27E-38 | GO:0042538 | hyperosmotic salinity response | 103 54 14261 67090 |
| 1.49E-38 | GO:0006606 | protein import into nucleus | 88 33 14276 67111 |
| 1.58E-38 | GO:0006816 | calcium ion transport | 84 28 14280 67116 |
| 1.36E-37 | GO:0042793 | transcription from plastid promoter | 69 13 14295 67131 |
| 1.72E-37 | GO:0000394 | RNA splicing, via endonucleolytic cleavage and ligation | 101 54 14263 67090 |
| 2.76E-37 | GO:0030244 | cellulose biosynthetic process | 113 74 14251 67070 |
| 4.56E-37 | GO:0006486 | protein glycosylation | 112 73 14252 67071 |
| 4.73E-37 | GO:0043085 | positive regulation of catalytic activity | 84 31 14280 67113 |
| 1.20E-36 | GO:0006950 | response to stress | 182 217 14182 66927 |
| 1.36E-36 | GO:0009909 | regulation of flower development | 159 166 14205 66978 |
| 2.55E-36 | GO:0009534 | chloroplast thylakoid | 71 17 14293 67127 |
| 2.76E-36 | GO:0006084 | acetyl-CoA metabolic process | 70 16 14294 67128 |
| 5.83E-36 | GO:0043069 | negative regulation of programmed cell death | 119 89 14245 67055 |
| 8.04E-36 | GO:0009965 | leaf morphogenesis | 122 95 14242 67049 |
| 8.51E-36 | GO:0010075 | regulation of meristem growth | 135 120 14229 67024 |
| 1.44E-35 | GO:0003676 | nucleic acid binding | 438 950 13926 66194 |
| 1.61E-35 | GO:0009579 | thylakoid | 58 6 14306 67138 |
| 1.34E-34 | GO:0051301 | cell division | 154 165 14210 66979 |
| 2.79E-34 | GO:0045454 | cell redox homeostasis | 99 60 14265 67084 |
| 3.34E-34 | GO:0016049 | cell growth | 97 57 14267 67087 |
| 3.37E-34 | GO:0043161 | proteasome-mediated ubiquitin-dependent protein catabolic process | 83 36 14281 67108 |
| 4.46E-34 | GO:0009863 | salicylic acid mediated signaling pathway | 87 42 14277 67102 |
| 4.97E-34 | GO:0016874 | ligase activity | 149 157 14215 66987 |
| 1.21E-33 | GO:0016757 | transferase activity, transferring glycosyl groups | 143 146 14221 66998 |
| 1.55E-33 | GO:0004722 | protein serine/threonine phosphatase activity | 92 51 14272 67093 |
| 1.86E-33 | GO:0005215 | transporter activity | 166 198 14198 66946 |
| 1.88E-33 | GO:0009266 | response to temperature stimulus | 75 27 14289 67117 |
| 3.01E-33 | GO:0006888 | ER to Golgi vesicle-mediated transport | 94 55 14270 67089 |
| 3.84E-33 | GO:0016853 | isomerase activity | 84 40 14280 67104 |
| 3.56E-32 | GO:0009693 | ethylene biosynthetic process | 87 47 14277 67097 |
| 3.67E-32 | GO:0016132 | brassinosteroid biosynthetic process | 83 41 14281 67103 |
| 5.96E-32 | GO:0009706 | chloroplast inner membrane | 71 25 14293 67119 |
| 6.21E-32 | GO:0009664 | plant-type cell wall organization | 105 78 14259 67066 |
| 7.58E-32 | GO:0019843 | rRNA binding | 65 18 14299 67126 |
| 8.58E-32 | GO:0003924 | GTPase activity | 108 84 14256 67060 |
| 1.16E-31 | GO:0007033 | vacuole organization | 62 15 14302 67129 |
| 1.37E-31 | GO:0016740 | transferase activity | 183 250 14181 66894 |
| 1.85E-31 | GO:0071555 | cell wall organization | 110 89 14254 67055 |
| 1.86E-31 | GO:0010218 | response to far red light | 74 30 14290 67114 |
| 2.94E-31 | GO:0005506 | iron ion binding | 237 394 14127 66750 |
| 5.02E-31 | GO:0000398 | mRNA splicing, via spliceosome | 81 41 14283 67103 |
| 5.20E-31 | GO:0050662 | coenzyme binding | 71 27 14293 67117 |
| 7.13E-31 | GO:0042542 | response to hydrogen peroxide | 99 71 14265 67073 |
| 8.21E-31 | GO:0007155 | cell adhesion | 100 73 14264 67071 |
| 1.24E-30 | GO:0010090 | trichome morphogenesis | 114 100 14250 67044 |
| 2.00E-30 | GO:0009637 | response to blue light | 73 31 14291 67113 |
| 2.00E-30 | GO:0045892 | negative regulation of transcription, DNA-templated | 102 78 14262 67066 |
| 2.17E-30 | GO:0006810 | transport | 115 103 14249 67041 |
| 2.97E-30 | GO:0032259 | methylation | 107 88 14257 67056 |
| 3.59E-30 | GO:0006626 | protein targeting to mitochondrion | 74 33 14290 67111 |
| 5.84E-30 | GO:0009630 | gravitropism | 109 93 14255 67051 |
| 8.68E-30 | GO:0048481 | ovule development | 90 59 14274 67085 |
