# Supplementary material for: Transcriptomic landscape of Pueraria lobata demonstrates potential for phytochemical study
Source: Front Plant Sci. 2015 Jun 22;6:426. doi: 10.3389/fpls.2015.00426 (PMC4476104; doi:10.3389/fpls.2015.00426)
Supplement: Supplementary file 5 [file Data_Sheet_5.DOCX]

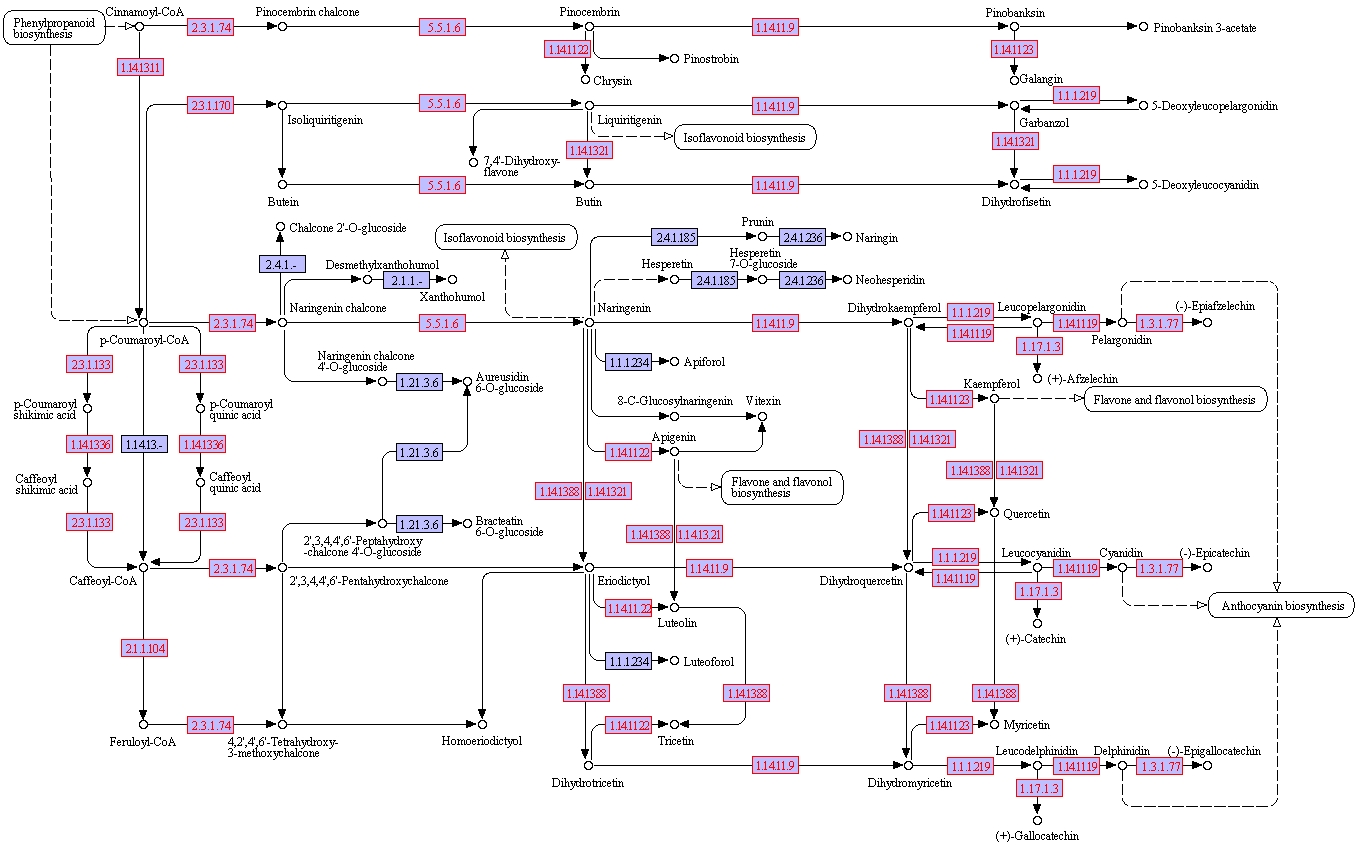


**Supplementary 5** Flavonoid biosynthetic pathway. The 19 enzyme commission numbers in red boxes were found in *P. lobata* dataset.


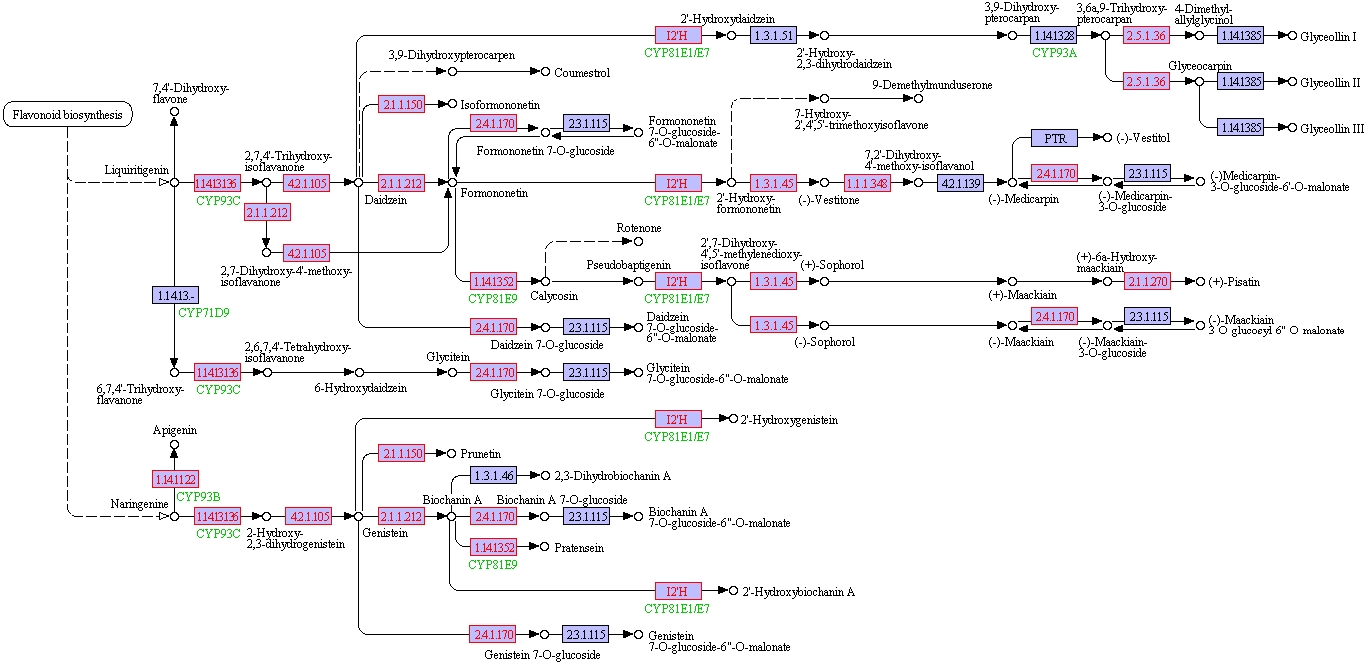


Isoflavonoid biosynthetic pathway. The 14 enzyme commission numbers in red boxes were found in *P. lobata* dataset.
