# Supplementary material for: Transcriptomic landscape of Pueraria lobata demonstrates potential for phytochemical study
Source: Front Plant Sci. 2015 Jun 22;6:426. doi: 10.3389/fpls.2015.00426 (PMC4476104; doi:10.3389/fpls.2015.00426)
Supplement: Supplementary file 6 [file Data_Sheet_6.DOCX]

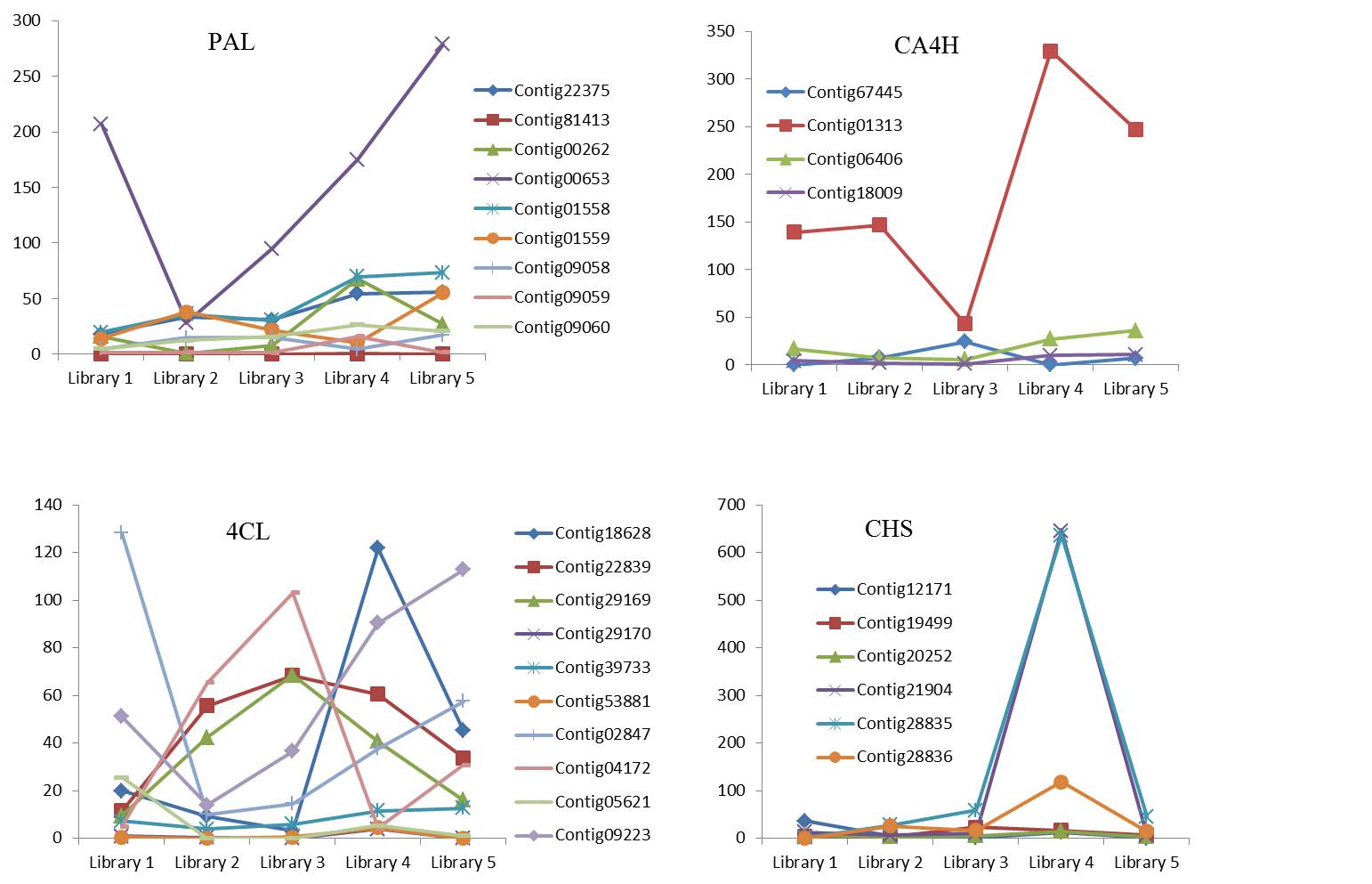


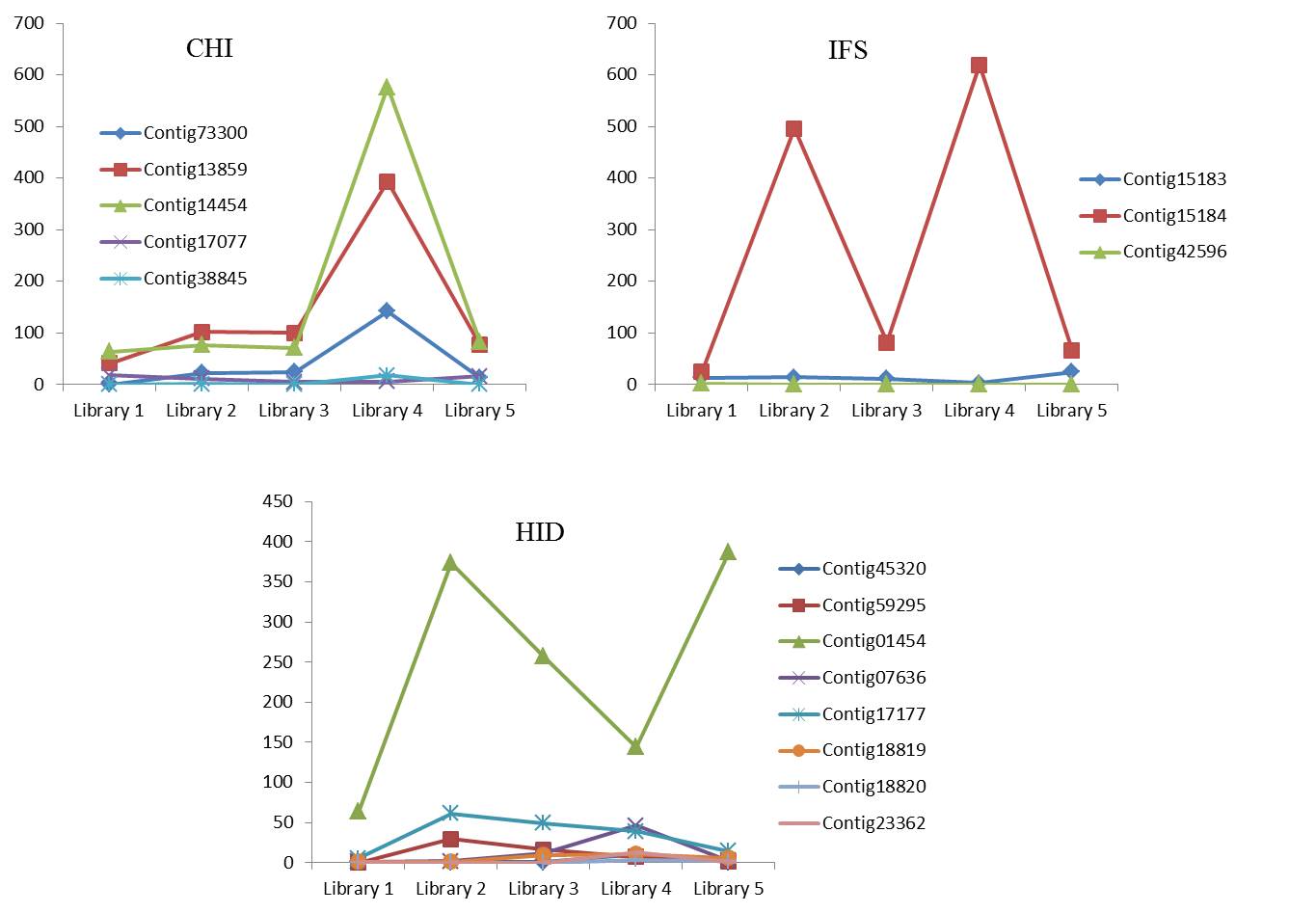


**Supplementary 6** The vertical axis indicates RPKM values of the annotated contigs involved in kudzu isoflavonoid biosynthesis while the horizontal axis shows five libraries in *P. lobata* dataset. PAL: Phenylalanine ammonia-lyase, CA4H: *Trans*-cinnamate 4-monooxygenase, 4CL: 4-coumarate-CoA ligase, CHS: 6'-deoxychalcone synthase, CHI: Chalcone isomerase, IFS: 2-hydroxyisoflavanone synthase, HID: 2-hydroxyisoflavanone dehydratase.
