# Supplementary material for: Transcriptomic landscape of Pueraria lobata demonstrates potential for phytochemical study
Source: Front Plant Sci. 2015 Jun 22;6:426. doi: 10.3389/fpls.2015.00426 (PMC4476104; doi:10.3389/fpls.2015.00426)
Supplement: Supplementary file 7 [file Data_Sheet_7.DOCX]

| Contig | Annotation | Forward primer | Reverse primer |
| --- | --- | --- | --- |
| 00518 | β-actin | TCCACTGGCATACAGAGACAAGA | GGCACCACTCAATCCCAAG |
| 21904 | CHS | AATGGCTGCCACCTTAGTCTCT | TCTTTTGTGGTAACTGTGCTGGTT |
| 14454 | CHI | GCAGTTTTCCATCACCTTCTTTG | GCTGGTTGAGACCCTTGACTTCT |
| 15184 | IFS | CTGTTGGGCCTCTGCACTTT | GTTCCCTTCGGACCTTACTGG |
| 01454 | HID | GCTTCCCACGCCAACAA | CCGCTGGTTTCACCTCCTAC |

**Supplementary 7** Primers designed for qRT-PCR experiment. CHS, chalcone synthase; CHI, chalcone isomerase;

IFS, 2-hydroxyisoflavanone synthase; HID, 2-hydroxyisoflavanone dehydratase.
