# Supplementary material for: Transcriptomic landscape of Pueraria lobata demonstrates potential for phytochemical study
Source: Front Plant Sci. 2015 Jun 22;6:426. doi: 10.3389/fpls.2015.00426 (PMC4476104; doi:10.3389/fpls.2015.00426)
Supplement: Supplementary file 8 [file Data_Sheet_8.DOCX]

**Supplementary 8** Contigs annotated as ABC transporter showing Pearson correlation coefficients to contig 01454 and contig 15184.

| Contig_ID | Coeffiecient_to_contig01454 | Coefficient_to_contig_15184 | Annotation |
| --- | --- | --- | --- |
| Contig00240 | -0.73 | -0.18 | ABC transporter D family member |
| Contig00241 | -0.77 | -0.5 | ABC transporter D family member |
| Contig02360 | 0.34 | -0.21 | Multidrug resistance protein ABC transporter family |
| Contig02621 | -0.56 | -0.72 | ABC transporter family protein |
| Contig03204 | 0.2 | -0.23 | Multidrug resistance protein ABC transporter family |
| Contig07356 | -0.63 | 0.6 | ABC transporter G family member |
| Contig07381 | -0.78 | -0.39 | ABC transporter family protein |
| Contig09681 | -0.78 | -0.41 | AAA ATPase; ABC transporter, transmembrane region, type 1 |
| Contig09727 | 0.05 | 0.58 | ABC transporter C family member |
| Contig10273 | -0.38 | 0.69 | ABC transporter G family member |
| Contig11124 | -0.79 | -0.44 | ABC transporter I family member |
| Contig16119 | -0.4 | 0.73 | Pleiotropic drug resistance ABC transporter family protein |
| Contig16951 | -0.97 | -0.24 | ABC transporter D family member |
| Contig17808 | -0.96 | -0.28 | ABC transporter D family member |
| Contig18533 | -0.91 | 0.06 | ABC transporter C family member |
| Contig22029 | -0.22 | 0.6 | ABC transporter C family member |
| Contig23310 | -0.72 | -0.47 | ABC transporter B family member |
| Contig27958 | -0.3 | -0.58 | ABC transporter C family member |
| Contig27959 | -0.38 | 0.58 | ABC transporter C family member |
| Contig31585 | -0.92 | -0.11 | ABC transporter C family member |
| Contig33504 | 0.05 | 0.37 | Multidrug resistance protein ABC transporter family |
| Contig33505 | 0.51 | 0.06 | Multidrug resistance protein ABC transporter family |
| Contig33602 | -0.65 | -0.5 | ABC transporter D family member |
| Contig36545 | 0.08 | 0.2 | ABC transporter B family member |
| Contig41300 | 0.5 | 0.22 | ABC transporter G family member |
| Contig41301 | 0.28 | -0.17 | ABC transporter G family member |
| Contig42074 | -0.06 | -0.5 | ABC transporter G family member |
| Contig44359 | -0.08 | -0.62 | ABC transporter B family member |
| Contig56331 | -0.72 | -0.47 | ABC transporter ATP-binding protein/permease |
| Contig58577 | -0.58 | -0.79 | ABC transporter B family member |
| Contig69143 | -0.4 | 0.73 | ABC transporter ATP-binding protein/permease |
| Contig69144 | -0.54 | -0.67 | ABC transporter ATP-binding protein/permease |
| Contig70145 | -0.4 | 0.73 | ABC transporter ATP-binding protein/permease |
| Contig73108 | 0.06 | 0.36 | Multidrug resistance protein ABC transporter family |
| Contig73572 | 0.43 | -0.12 | Multidrug resistance protein ABC transporter family (Fragment) |
| Contig74670 | -0.34 | 0.69 | ABC transporter G family member |
| Contig76477 | 0.26 | 0.89 | Multidrug resistance protein ABC transporter family (Fragment) |
| Contig76628 | 0.43 | -0.12 | Multidrug resistance protein ABC transporter family |
| Contig79472 | -0.4 | 0.73 | ABC transporter B family member |
| Contig80235 | -0.4 | 0.73 | ABC transporter B family member |
| Contig81715 | 0.02 | 1 | ABC transporter B family member |
