# Supplementary material for: Transcriptomic landscape of Pueraria lobata demonstrates potential for phytochemical study
Source: Front Plant Sci. 2015 Jun 22;6:426. doi: 10.3389/fpls.2015.00426 (PMC4476104; doi:10.3389/fpls.2015.00426)
Supplement: Supplementary file 9 [file Data_Sheet_9.DOCX]

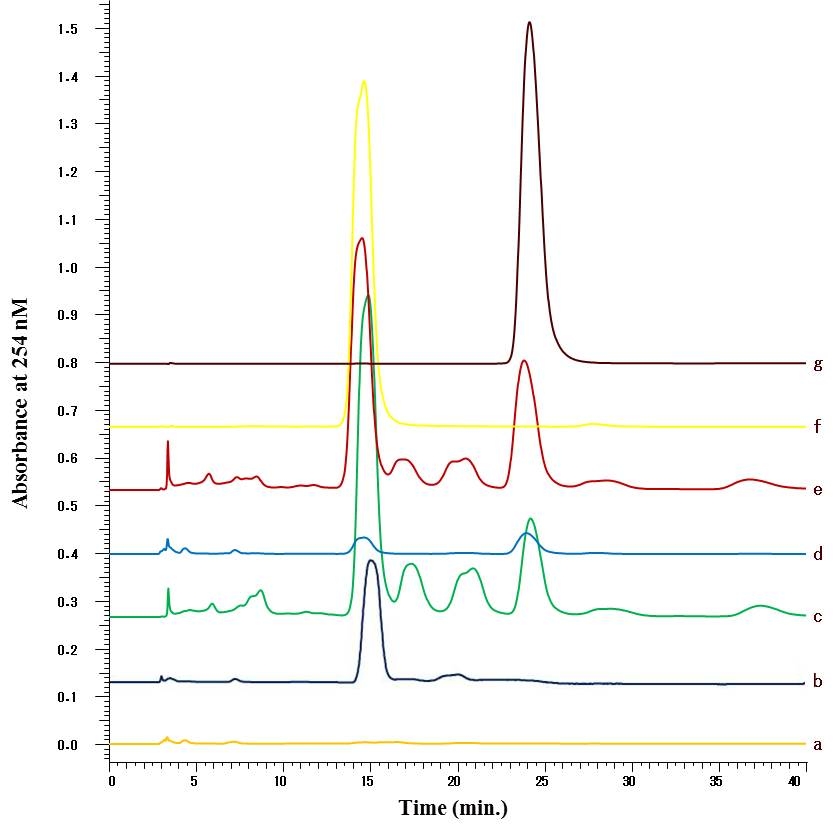


**Supplementary 9** HPLC profiles of extracts from 5 tissues of *P. lobata*. a Leaf, b Stem, c Mature root, d Young root, e Root vascular cylinder, f Puerarin standard, g Daidzin standard.
